# Barriers to access and permanence at the university: a point of view

**DOI:** 10.31744/einstein_journal/2021ED6447

**Published:** 2021-12-15

**Authors:** Isabel Cristina Torres-Patiño, Cristhiam Mauricio Rojas-Hernandez, Herney Andres García-Perdomo

**Affiliations:** 1 Universidad del Valle Cali VA Colombia Universidad del Valle, Cali, VA, Colombia.; 2 University of Texas MD Anderson Cancer Center Houston TX United States University of Texas MD Anderson Cancer Center, Houston, TX, United States.

Currently, adolescents need to access higher degree education if they want to have a successful professional career. Unfortunately, even if they want, some of them cannot easily access or pursue it. Different reasons exist regarding this phenomenon such as financial constraints, cultural issues, or lack of available positions at public and/or state universities. This issue is not exclusive of developing countries, and each country has different nuances of the problem. In the United States, one-third of the population is below the poverty line, but just 6% of medical students come from these families.^( 1 )^ In Latin America the coverage, meaning the rate of college enrollment, passed from 7% in 1970 to 41% (21 millions of students) in 2010. These rates mean that more than 30 years was necessary to achieve a coverage around 50%.^( 2 )^

Unfortunately, this issue is far from being solved. The most affected populations are the poor given that governments have been incapable of closing the socioeconomic gap that let many adolescents without the opportunity to access higher degree education and succeed in a career. Furthermore, the actual situation of social distancing and financial crisis due to the coronavirus disease 2019 (COVID-19) pandemic, may lead to an increase in the socioeconomic gap, considering that only few would bel able to afford higher degree education in colleges and universities.^( 2 )^ Thus, it is necessary to keep studying the situation in different countries from a variety of angles, and also recognizing the different barriers to access higher degree education, and pursuing a professional and academic career. In this editorial, barriers are defined as any obstacle that prevents the population from access undergraduate studies. Data included in this study cover the rate of enrollment at higher education only.

## THE CURRENT SITUATION IN AMERICA AND EUROPE

Latin America improved access to higher education at universities by using different strategies such as funding for public universities, scholarships for students, and educational loans.^( 2 )^ Ecuador, after had been the country with the least cover and educational quality,^( 2 )^ adopted these strategies to rose the access to university by offering 55.56% and the gross and net rate of enrollment to 31.86% and 21.23%, respectively. Likewise, Argentina is currently known for its high quality system and unrestricted free access to education for Argentinian nationals.^( 3 )^ In contrast, however, countries as Colombia, Ecuador, and Brazil although offer free education, the access to it is restricted due to the lack of budget. For this reason, universities have been adopted an admission test to select candidates for open positions, and this system has further reinforced inequalities, given that better-prepared students often do not belong to low-income families.^( 2 )^

Moreover, even if access to higher education could be improved in the Americas, the number of students that obtain a degree would be very low. In Latin America the rates of college dropout are 57%, and this ranges from 82% in countries like Guatemala to 40% in countries like Argentina. In developed countries such as Spain and the United States, this rate is not so different, dropout is around 30% to 50%. In European countries such as Germany or Finland, the college dropout is about 10% to 25%.^( 4 )^While no huge differences exist in dropout rates between North- and Latin America; there are important differences in the access to higher education. These differences can be partially explained by other factors, *e.g.,* the investment by large company in higher education (universities, colleges) that is significant higher in developed countries than their developing counterparts.^( 2 )^ During the great recession, financial support declined and many colleges and universities shifted from offering need-based scholarships to merit-based scholarships. In addition, they reduced support services to student, which may have impact on those from disadvantaged families.^( 1 )^

## PRINCIPAL BARRIERS

The main obstacle to access higher degree education is the economic barrier, which is presents in two ways: the low income in the population and the lack of investment in education by the government. Poverty does not only restrict the students’ capacity to pay tuition fees, this also keep them from being prepared for living and growing throughout the educational experience.^( 1 )^ Actually, high cost of tuition has been recognized among the minorities as the main reason that difficult the access to education.^( 5 )^ This shortage impacts the student’s life and conceals them from taking the full advantage of the university, even if they are at the best classroom. Poverty may triggers different factors such as poor health, housing instability, crime, and inadequate pre-literacy experiences.^( 1 )^ The household socioeconomic status has been described as the most consistent predictor for preventing the access to good education in childhood.^( 1 )^ In other words, the social inequality does not only affect the access to college but to the quality of education throughout all the steps of schooling. This is evident during higher education when students from disadvantaged socioeconomic backgrounds are challenged by certain standard levels of general literacy, and this limitation may ultimately make them to end up dropping out from college.^( 3 )^

This socioeconomic gap also affects adolescents’ life given that if a student is the first household member to go to the university, he/she probably will not graduate. As a matter of fact, a 90% rate has been reported on this kind of students that dropout from college.^( 1 )^ Students whose parents went to university felt highly motivated to follow their inheritance. However, students from disadvantaged families are less motivated consider that they do not have anyone to guide them throughout the academic path.^( 3 )^ These are the main reasons that poverty may keep students from accessing and pursuing an academic career. The paragraphs will discuss the role of the government to address this problem.

The second issue is the lack of investment in education by government. There is a lack of coverage in public universities due to the insufficient budget that makes the private sector to take control of education. Also, the agencies responsible to regulate education at these institutions do control them, for this reason consequences are inequality and higher tuition fees. Even if those constraints could be solved, the government needs to supply services to student at all universities to support their career path.^( 2 )^ For this, in 1998 the United Nations Educational, Scientific and Cultural (UNESCO) has declared that access to higher education must be guaranteed for all the population, regardless of race, ethnicity, or socioeconomic status.^( 2 )^

The limitation stated above shows that most of the populations are at disadvantage to pursue a professional career. However, there are other factors such as belonging to minority groups that limit, even more, the access to higher education. This limitation is mainly related to the insufficient number of slots granted to the minorities. An example of this situation is the *Universidad Nacional de Colombia* , where 2% of the slots are adjudge to the indigenous groups, but they represent 3.43% of the country population.^( 6 )^ In addition, the admission process involves the need of documents to prove that applicants belong to one of the minority groups. Those documents might be confusing to complete and difficult to send because they may require access to internet services, which often lacks in those regions. The universities do not know how respect these minorities differences or how to act when they become regular students. Consequently, the dropout of this group is almost immediate, and most of them finish the first year of tuition only. These rates show that a college career is more than complete assignments, it is part of social and cultural organization.

Gender differences have been also recognized as barrier to access higher education. The universities have been unfavorable places for women, and their participation in higher education went through different struggles since the beginning of the XIX century. The Americas and Central Asia, and Europe in 2009 reported the Gender Parity Index (GPI) as 1.08 in favor of women according to UNESCO. Unfortunately, this index does not reflect the numbers of those moving from master’s degree to PhD. The decline is even more in the academic appointments and research, thus the proportion between man and women in the research appointments is 71% to 29% and the percentage of tenures is 26,9% in Mexico. Also, the women represent the 42.2% of the professorship in that country.^( 7 )^ Additionally, the permanence of women inside and outside of the university is threatened for the sexual harassment.^( 7 )^

## ADVANCES FOR SOLUTIONS

Government must guarantee access and permanence at university, and all individuals must have to the same opportunities, which may reduce the gap to access for schooling.^( 2 )^ Consequently, Jara et al., discussed that “ *La inclusión educativa debe ser una prioridad estatal, fomentada desde las normativas constitucionales, leyes, reglamentos y ordenanzas, con el propósito de reducir barreras que excluyen al ser humano de una de las necesidades sociales fundamentales como es la educación.* ”^( 8 )^ According to this, Sinchi et al., proposed as solution the diversification of the funding sources with the budget rationale, offering of unrestricted access for all the population, and converting of all the possible expenses that the student from disadvantages family would need throughout the career.^( 2 )^ Also, the governors must take control of educational initiatives. For example, Sinchi et al., described the poor quality of the education at all levels, but only in 2010 the “organic law of high education” was established. This has benefited the access and the improved the education quality at universities.^( 2 )^

Universities have been implemented other kinds of solutions, such as scholarships for preventing the dropouts. Both public and private universities use this strategy for some students based on their economic situations, high grades, sporting merit, and presence of disabilities. Another solution is the credit as an attempt to solve the problem for those families that cannot afford a private university. Student is committed to pay this loan in the future.^( 2 )^ However, the negative side is that he/she starts working life with a debt that could last for a long time, and may prevent them to advance and improve the socioeconomic status. Even if the economic barrier is the most significant one for accessing university, it is also necessary that the students have an institutional involvement in pursuing the career. One of the strategies that can help them is to explore available resources such as information centers or mentorship opportunities.^( 3 )^

There are strategies to improve access and permanence in academic career for minorities. An example is the *“Admisión de Estudiantes de Pregrado por Programas de Admisión Especial* (PAES)” program that started in 1986 at the *Universidad Nacional de Colombia* . The program included only seven students that remained for the first year only; accordingly, it was necessary to stake out the program and include a different process for permanence, recognizing their contributions to ancestral knowledge and gave them an economic incentive that allowed them to stay in the city. In addition, they received support to basic science mentorship, lodging and medical service.^( 6 )^

With the goal to ameliorate gender inequality at universities, some institutions have formulated policies and guidelines to achieve equality, including the development of campaigns, programs and policies. Important actions that have been implemented by universities are the establishment of protocols and instances to report complaints of discriminations, bullying, harassment and other ways of gender violence.^( 7 )^

## CONCLUSION

Barriers to access and permanence in a career are diverse and affect differently each population. Furthermore, the main barrier that the people may face related to socioeconomic status. This is a complex situation and no single solution exists, and this may include the intervention of many actors like government, universities, large companies, among others. The best resources for solving are regulations that protect education at all the levels, and for all the population, and the guarantee of same quality of literacy and opportunity. For this reason, the government must recognize education as a priority.

Although the problem still exists, there have been advances in the last 50 years because now some governments have recognized that education is a necessary priority. Many organizations are trying to solve the access issue in the best way they can, and now, fortunately, the rates of access have improved.
