# Supplementary material for: Barriers to access and permanence at the university: a point of view
Source: Einstein (Sao Paulo). 2021 Dec 15;19:eED6447. doi: 10.31744/einstein_journal/2021ED6447 (PMC8687642; doi:10.31744/einstein_journal/2021ED6447)
Supplement: Translation

# Translation: Barreiras de acesso e permanência na universidade: um olhar

**Authors:** Isabel Cristina Torres-Patiño, Cristhiam Mauricio Rojas-Hernandez, Herney Andres García-Perdomo

**Affiliations:** 1 Colombia Universidad del Valle, Cali, VA, Colombia.; 2 United States University of Texas MD Anderson Cancer Center, Houston, TX, United States.

Atualmente, os adolescentes que almejam uma carreira profissional de sucesso precisam ter acesso à educação superior. Infelizmente, mesmo quando pretendido, o acesso ou a permanência no ensino superior não são tarefas fáceis. Há diversos motivos para esse fenômeno, como limitações financeiras, questões culturais ou falta vagas nas universidades públicas. Esse problema não é exclusivo dos países em desenvolvimento, e cada país possui particularidades diferentes. Nos Estados Unidos, um terço da população está abaixo da linha da pobreza, e apenas 6% dos estudantes de medicina são originários dessas famílias.^( 1 )^Na América Latina, o acesso à faculdade/universidade, isto é, o número de inscrições no ensino superior, passou de 7%, em 1970, para 41%, atingindo o total de 21 milhões de estudantes em 2021. Esse número sugere que foram necessários mais de 30 anos para atingir acesso à universidade próximo a 50%.^( 2 )^

Contudo, o problema está longe de ser resolvido. As populações mais afetadas são as pobres, dada a incapacidade dos governos de preencher a lacuna socioeconômica que deixou muitos jovens sem acesso à educação superior e possível sucesso na carreira. Além disso, a situação atual de distanciamento social e crise financeira devido à pandemia da doença pelo coronavírus 2019 (COVID-19) pode levar ao aumento da lacuna socioeconômica, sendo que poucos podem pagar por educação superior particular.^( 2 )^Com isso, há necessidade em continuar a investigação sobre a situação nos diferentes países, a partir dos mais diversos ângulos, também como de reconhecer as diferentes barreiras de acesso à educação superior e à carreira acadêmica ou profissional. Neste editorial, define-se como barreira a existência de qualquer obstáculo que limita a população em acessar o curso de graduação. Os dados informados incluem apenas números de inscrições relacionadas ao ensino superior.

## A SITUAÇÃO ATUAL NAS AMÉRICAS E NA EUROPA

A América Latina melhorou o acesso à educação superior (em universidade) por meio da adoção de estratégias diferentes, como investimentos em universidades públicas, bolsas para estudantes e acesso a crédito para pagamento de educação superior.^( 2 )^Por meio de tais ações, o Equador aumentou o acesso ao ensino superior para 55,56%, quantidade e taxa de inscrição para 31,86% e 21,23%, respectivamente, após ter sido considerado o país com menor taxa de acesso e qualidade educacional.^( 2 )^A Argentina, hoje reconhecida por seu sistema de alta qualidade e de acesso gratuito irrestrito a sua população a educação, também melhorou seus índices.^( 3 )^Porém, na contramão, países como Colômbia, Equador e Brasil atualmente oferecem educação gratuita, mas não garantem acesso a toda população, devido à falta de orçamento. Por esse motivo, as universidades possuem um exame de admissão para alocar as vagas gratuitas, que reforça essas diferenças, já que os melhores e mais bem preparados estudantes nem sempre pertencem às famílias de baixa renda.^( 2 )^

Além disso, mesmo se o acesso ao ensino superior fosse melhorado nas Américas, o número de estudantes que obtém formação superior seria muito baixo. Na América Latina, as taxas de evasão do ensino superior somam 57%. Os níveis mais altos podem chegar a 82% em países como a Guatemala, e os mais baixos a 40% em países como Argentina. Em países desenvolvidos, como Espanha e Estados Unidos, a porcentagem de evasão não é tão diferente: cerca de 30% a 50%. Em países europeus, como Alemanha e Finlândia, essa mesma taxa varia de 10% a 25%.^( 4 )^Enquanto parece não haver grandes diferenças nas taxas de evasão entre América do Norte e América Latina, existem diferenças importantes em relação ao acesso à educação superior. Essas diferenças podem ser explicadas parcialmente por outros fatores, como, por exemplo, o investimento de grandes empresas no ensino superior (universidade e faculdades) ser mais significante em países desenvolvidos do que em nações em desenvolvimento.^( 2 )^Durante a grande recessão, o investimento foi reduzido, e muitas faculdades e universidades mudaram o sistema de bolsa baseada em necessidade para um sistema de bolsa baseada em mérito. Além disso, os serviços de apoio aos estudantes também foram reduzidos, afetando principalmente aqueles provenientes de famílias carentes.^( 1 )^

## PRINCIPAIS BARREIRAS

Entre os principais obstáculos ao acesso à educação superior está a questão econômica, que se apresenta de duas formas: a baixa renda da população e a falta de investimento em educação pelo governo. A pobreza não é restrita somente à capacidade de os estudantes de pagar mensalidades, mas também de estarem preparados e se manterem durante a experiência educacional.^( 1 )^De fato, os altos custos das mensalidades têm sido reconhecidos, na minorias dos casos, como o principal fator que dificulta o acesso.^( 5 )^Essa limitação impacta a vida do estudante e pode ser um fator impeditivo ao aproveitamento integral na vida universitária, mesmo àqueles que estão nas melhores salas de aula. A falta de recursos financeiros pode ser responsável pelo desencadeamento de diferentes fatores, como problemas de saúde, instabilidade de moradia, criminalidade e experiências inadequadas pré-letramento.^( 1 )^A situação socioeconômica tem sido descrita como o preditor mais consistente para atingir uma boa educação na infância.^( 1 )^Em outras palavras, as inequidades sociais não apenas afetam o acesso ao ensino superior, mas a qualidade da educação ao longo de todos os estágio da educação. É evidente que, durante a faculdade, quando os estudantes em desvantagens socioeconômicas são desafiados por certos níveis de padrões de letramento geral, eles acabem desistindo da educação superior.^( 3 )^

Essa limitação socioeconômica também afeta a vida dos adolescentes. Caso o estudante seja o primeiro membro da família a ir para universidade, ele provavelmente não concluirá a graduação. De fato, 90% desses estudantes interrompem os estudos.^( 1 )^Filhos de pais que frequentaram a universidade se sentem mais motivados em seguir o mesmo caminho, diferentemente de estudantes provenientes de famílias em desvantagens econômicas, que não possuem ninguém para auxiliá-los a trilhar o caminho acadêmico.^( 3 )^Esse é um dos principais fatores que mantêm os estudantes longe de acessar e cursar a carreira acadêmica. A partir de agora, vamos discutir o papel do governo nessas questões.

Há outro ponto ligado à falta de investimento dos governos em educação: a falta de oferta suficiente em universidades públicas devido ao orçamento insuficiente, fazendo com que o setor privado controle a educação. Também, as agências responsáveis falham no controle dessas instituições, levando à qualidade insuficiente e a alto custos. Mesmo se essas limitações fossem resolvidas, o governo precisaria disponibilizar os serviços que os estudantes precisarão em sua carreira na universidade.^( 2 )^Portanto, como declarado em 1998 pela *United Nations Educational, Scientific and Cultural Organization* (UNESCO), o acesso à educação superior deve ser garantido para toda a população, independente de raça, etnia ou situação socioeconômica.^( 2 )^

Esses itens mostram que a maioria das populações está em desvantagem para acessar a carreira profissional. Porém, há outros fatores, como o pertencimento às minorias que têm acesso ainda mais limitado à educação superior. Isso ocorre principalmente em relação ao número reduzido de vagas reservadas às minorias. Um exemplo é da *Universidad Nacional de Colombia* , onde 2% dos estudantes são considerados indígenas, mas eles representam 3,43% da população do país.^( 6 )^Também, para admissão, há necessidade do preenchimento de documentação que comprove que o candidato pertence a um desses grupos minoritários. Contudo, essa documentação pode ser confusa, e o preenchimento e envio podem ser dificultados, já que requerem acesso a *internet* – serviço que é insuficiente nessas regiões.

As universidades desconhecem as regras para respeitar as diferenças ou mesmo como agir no caso da admissão de estudantes provenientes de tais grupos minoritários. Consequentemente, a evasão desses grupos é quase imediata, e a maioria deles completa somente o primeiro ano do curso. Tal fato mostra que o curso superior é mais do que uma atividade, isto é, trata-se uma forma de integrar uma organização cultural e social.

As diferenças têm sido reconhecidas como barreira para acessar à educação superior. As universidades são consideradas locais não favoráveis para mulheres, e a participação delas na educação superior passou por diferentes limitações, desde do início do século 19. Isso permitiu que, em 2009, o Índice de Igualdade de Gênero (IIG) na Europa, Américas e Ásia Central foi de 1,08 em favor das mulheres, de acordo com a UNESCO. Infelizmente, isso não reflete os números daqueles que prosseguiram para o Mestrado e Doutorado. O declínio é ainda maior em posições acadêmicas e em pesquisa. A proporção entre homens e mulheres em pesquisa é de 71% para 29%, e a percentagem de professoras com dedicação exclusiva é de 26,9% no México. Também, naquele país, as mulheres representam 42,2% dos profissionais.^( 7 )^Além disso, a permanência dentro e fora da universidade das mulheres é ameaçada por conta do assédio sexual.^( 7 )^

## AVANÇOS PARA SOLUÇÕES

O governo deve garantir acesso e permanência na universidade, e todas as pessoas devem ter as mesmas oportunidades, com o objetivo de reduzir a lacuna no acesso à educação.^( 2 )^Consequentemente, Jara et al., afirmaram que “ *La inclusión educativa debe ser una prioridad estatal, fomentada desde las normativas constitucionales, leyes, reglamentos y ordenanzas, con el propósito de reducir barreras que excluyen al ser humano de una de las necesidades sociales fundamentales como es la educación.* ”^( 8 )^Com isso, Sinchi et al., propuseram como solução a diversificação da fonte de financiamento como justificativa para o orçamento e, desta forma, oferecer acesso irrestrito para todas as populações e cobrir todos os possíveis gastos que o estudante de famílias em desvantagens precisaria ao longo da carreira.^( 2 )^Também, os governos devem ter responsabilidade para controlar a educação. Por exemplo, Sinchi et al., descreveu baixa qualidade da educação em todos os níveis, contudo, apenas em 2010, a “lei orgânica de educação superior” foi estabelecida. Isso beneficiou o acesso e a melhora da qualidade da educação nas universidades.^( 2 )^

As universidades têm dado outros tipos de soluções, como bolsa de estudos para prevenir a evasão. Tanto universidades públicas quanto privadas usam essa estratégia para alguns estudantes, de acordo com a situação econômica, e também se baseiam em notas mais altas, mérito esportivo ou para estudantes com necessidades especiais. Outra solução é o crédito, que é uma tentativa de resolver o problema entre as famílias que não podem pagar uma universidade privada. O estudante se compromete em pagar o empréstimo no futuro.^( 2 )^Contudo, o indivíduo começa sua vida profissional com um débito que pode levar muito tempo para quitar. Dessa forma, isso o previne a avançar e sair da linha da pobreza. Mesmo com as barreiras econômicas sendo um dos fatores mais significante para acesso à universidade, há também a necessidade em garantir que os estudantes possam ser capazes de progredir em uma carreira. Uma das estratégias que pode ajudá-los é o uso de recursos disponíveis, como busca de informações em centros de informação ou procura por mentores.^( 3 )^

Existem estratégias para melhorar o acesso e a permanência na carreira acadêmica das minorias. Um exemplo é a *“Admisión de estudiantes de pregrado por Programas de Admisión Especial* (PAES).” Esse programa foi iniciado em 1986 na *Universidad Nacional de Colombia* , com a inclusão de apenas sete estudantes que permaneceram somente no primeiro ano na universidade. Em certa medida, foi necessário acompanhar o programa e incluir um processo diferente para permanência deles, reconhecendo suas contribuições ao conhecimento e disponibilizando incentivo econômico que permitisse que os estudantes continuassem na cidade. Além disso, os indivíduos receberam apoio, como mentoria em ciências básica, hospedagem e serviço de saúde.^( 6 )^

Com o objetivo de melhorar a desigualdade de gênero nas universidades, algumas instituições têm adotado políticas e diretrizes formuladas para atingir tal igualdade, incluindo o desenvolvimento de campanhas, programas e políticas. Um passo importante nas universidades é a implementação de protocolos e organismos para registro de denúncias de discriminação, *bullying* , assédio e outras formas de violência de gênero.^( 7 )^

## CONCLUSÃO

As barreiras para acessar e permanecer na carreira são diversas e afetam cada população de forma diferente. A principal barreira que os indivíduos enfrentam é a socioeconômica. A questão é complexa, e não há uma única solução, sendo que há necessidade de intervenção de muitos atores, como o governo, as universidades, as grandes empresas, entre outros. O melhor recurso para resolver a questão é o estabelecimento de diretrizes que protejam a educação em todos os níveis e garantam acesso a toda a população ao ensino de forma igualitária à qualidade de letramento e à oportunidade para desenvolvimento. Portanto, os governos devem reconhecer essa educação como questão prioritária.

Atualmente, o problema permanece, e avanços têm sido feito nos últimos 50 anos em termos de alguns governos reconhecerem essa necessidade. A maioria das organizações têm tentando resolver a questão de acesso da melhor forma que podem, e hoje, felizmente, as taxas de acesso ao ensino superior têm crescido.
